# Effects of group music sessions on cognitive and psychological functions in healthy older adults

**DOI:** 10.3389/fragi.2025.1513359

**Published:** 2025-02-10

**Authors:** Takamitsu Shinada, Michio Takahashi, Akari Uno, Keishi Soga, Yasuyuki Taki

**Affiliations:** ^1^ Smart Aging Research Center, Tohoku University, Sendai, Japan; ^2^ Department of Aging Research and Geriatric Medicine, Institute of Development, Aging and Cancer, Tohoku University, Sendai, Japan

**Keywords:** music, session, aging, cognitive function, mood

## Abstract

**Introduction:**

With the rapid aging of the population worldwide and the prevalence of dementia and mental health problems among older adults, it is important to extend healthy life expectancy by maintaining brain and mental health. Playing musical instruments, which requires the integration of auditory, visual, and somatosensory functions, is considered an effective way to prevent the development of dementia. However, the effectiveness of group (band) music sessions in healthy older adults has not been investigated. Our purpose, therefore, was to investigate the effects of group music sessions on cognitive and psychological functions among healthy older adults.

**Methods:**

In this open-label randomized controlled trial, participants aged 65–74, who had no musical experience, were randomly assigned to either the intervention or control group. The intervention group received in weekly 90-minute sessions with the instrument for 16 weeks. The control group received no intervention.

**Results:**

The results showed that the Mini-Mental State Examination (MMSE) total score and the Wechsler Memory Scale Logical Memory Ⅱ (WMS-LM Ⅱ) score improved significantly, and the Vigor–Activity subscale score of the Profile of Mood States 2nd Edition (POMS 2) tended to improve.

**Discussion:**

These findings indicated that group music sessions have a potentially beneficial effect for maintaining and improving cognitive and psychological functions in healthy older adults.

## 1 Introduction

Developed countries are aging, with the proportion of the population over 65 years standing at 17.5% worldwide (Padeiro et al., 2023) and at over 30% in Japan (Otsu and Shibayama, 2022). Accordingly, the number of people with dementia worldwide was projected to increase to 57.4 million in 2019, further rising to 152.8 million in 2050 (Nichols et al., 2022). In Japan, the total annual financial loss due to dementia exceeds approximately 12 trillion yen ($180 billion), placing a heavy burden on the nation’s finances (Ikeda et al., 2021). As Japan’s population ages, healthy life expectancy is increasing along with average life expectancy, and although the gap is narrowing, it is still large (about nine years for men and 12 years for women) (Ministry of Health, Labour and Welfare, 2019). Extending healthy life expectancy, defined as “the period during which people can live without having their daily lives restricted by health problems,” is necessary to realize a vibrant society in which each person can live a healthy and enriched life and to make the social security system sustainable. In addition, retired older adults have a lot of leisure time, and it is believed that spending time meaningfully and happily doing what each enjoys can improve their sense of well-being and extend their healthy life expectancy (Minagawa and Saito, 2023).

Many activities contribute to dementia prevention (Asteasu et al., 2017; Klimova et al., 2020; Marioni et al., 2015; Piolatto et al., 2022); music is one of them (Dingle et al., 2021). In addition to listening to music, playing has been shown to be effective in improving cognitive function (Gómez-Gallego et al., 2021). As many parts of the brain are activated in an integrated manner during performance, using multiple senses—auditory, visual, and tactile—simultaneously (Zatorre et al., 2007; Lappe et al., 2008), regularly playing musical instruments with such a multimodal component contributes to improved cognitive function and maintenance of gray matter volume in the frontal and temporal regions in old age (Böttcher et al., 2021; Böttcher et al., 2022). For example, in a cohort study of 1,570 older adults, experience playing a musical instrument was associated with significant improvements in cognitive function (working memory and executive function) (Vetere et al., 2024). In an intervention study of healthy older adults unfamiliar with music, piano lessons requiring bimanual coordination improved executive function and working memory (Bugos et al., 2007; Bugos, 2019). In healthy older adults with no musical experience, four months of keyboard harmonica lessons significantly improved memory performance on a test measuring verbal recall (Guo et al., 2021). Furthermore, the significant improvement in memory performance was associated with a significant reduction in the level of functional connectivity (FC) between the left putamen seed and right superior temporal gyrus (lPu-rSTG) in functional magnetic resonance imaging, suggesting improved neural efficiency. Working memory (WM) training has been reported to reduce resting-FC between medial prefrontal cortex and the right posterior parietal cortex/right lateral prefrontal cortex, which are key nodes of the external attention system in WM (Takeuchi et al., 2013). Patterns of activation resulting from cognitive training have been suggested to include patterns of increased activation, reduced activation, and a combination of increased and reduced activation, and among these, reduced activation has been attributed primarily to increased neural efficiency (Buschkuehl et al., 2012). This concept is closely related to the “neural efficiency theory” proposed by Haier (Haier et al., 1988; Haier et al., 1992a; Haier et al., 1992b). This theory assumes that when participants are performing a task well, fewer neurons are recruited than when they are not performing well. Therefore, less proficient performance could activate brain circuits that are unimportant or even detrimental to task performance. Previous studies have shown that WM training reduced BOLD responses in brain regions related to WM during WM performance. At the same performance level, reduced activation was observed, suggesting that WM training may increase neural efficiency (Brehmer et al., 2011; Vartanian et al., 2013; Heinzel et al., 2014).

In addition to dementia, mental health is another important issue that needs to be addressed in an aging society (Phongsavan et al., 2013). Aging potentially increases the risk of mental health problems. In areas with large older populations, a higher percentage of people experience depression and other mental illnesses, and mental illness among older adults is expected to increase (Zenebe et al., 2021). Depression is also considered a risk factor for dementia (Cantón-Habas et al., 2020; Zhang et al., 2021), and maintaining psychological health may help prevent dementia (Johansson et al., 2010). Music interventions have positive effects on the mental health of older adults. In healthy older adults, listening to music of their choice for 30 min each week for 4 weeks improved geriatric depression scores (Chan et al., 2010). Depressive symptoms improved when older adults with depression received active music therapy (in which participants played percussion instruments) once every two weeks for 60 min, for a total of 20 sessions (Erkkilä et al., 2011). Furthermore, healthy older participants showed improvements in mood state and quality of life after four months of piano lessons (Seinfeld et al., 2013). It is also known that older adults who are more likely to use music for emotional aspects, such as listening to music to make themselves feel better or for enjoyment, have higher subjective well-being (Wang and Suh, 2024).

Unlike playing a musical instrument alone, group music sessions provide a sense of togetherness, playing in tune and rhythm with others. Music sessions have been incorporated as loop work for patients with dementia, and there have been improvements in psychological state, such as anxiety levels, after six weeks of percussion group sessions among older adults with dementia (Sung et al., 2012). A three-month drum session program for patients with dementia, including mild cognitive impairment, residing in a nursing home showed significant improvement in Mini-Mental State Examination and Frontal Assessment Battery scores and improved cognitive function (Miyazaki et al., 2020). In healthy older adults, self-efficacy and processing speed improved after 8 weeks of percussion (mallet) group sessions (Bugos and Cooper, 2019). Lessons involving 10 weeks of piano ensembles have been reported to improve executive function in healthy older adults (MacRitchie et al., 2020). Sixteen weeks of piano lessons, including two group training sessions per week of 90 min each, improved general and musical self-efficacy (Bugos and Wang, 2022). In addition, physiological effects of enhanced immune function have been found in older adults who have participated in drumming group sessions (Koyama et al., 2009). However, to the best of our knowledge, there are no studies on the cognitive and psychological effects of group (band) music sessions with healthy older adults who have no previous experience with musical instruments. In addition, participating in group music sessions where each person plays a different instrument is expected to significantly enhance cognitive function. This improvement arises from the complex cognitive processing required to listen to others while simultaneously performing one’s own music.

## 2 Methods

### 2.1 Participants and experimental design

**FIGURE 1:**
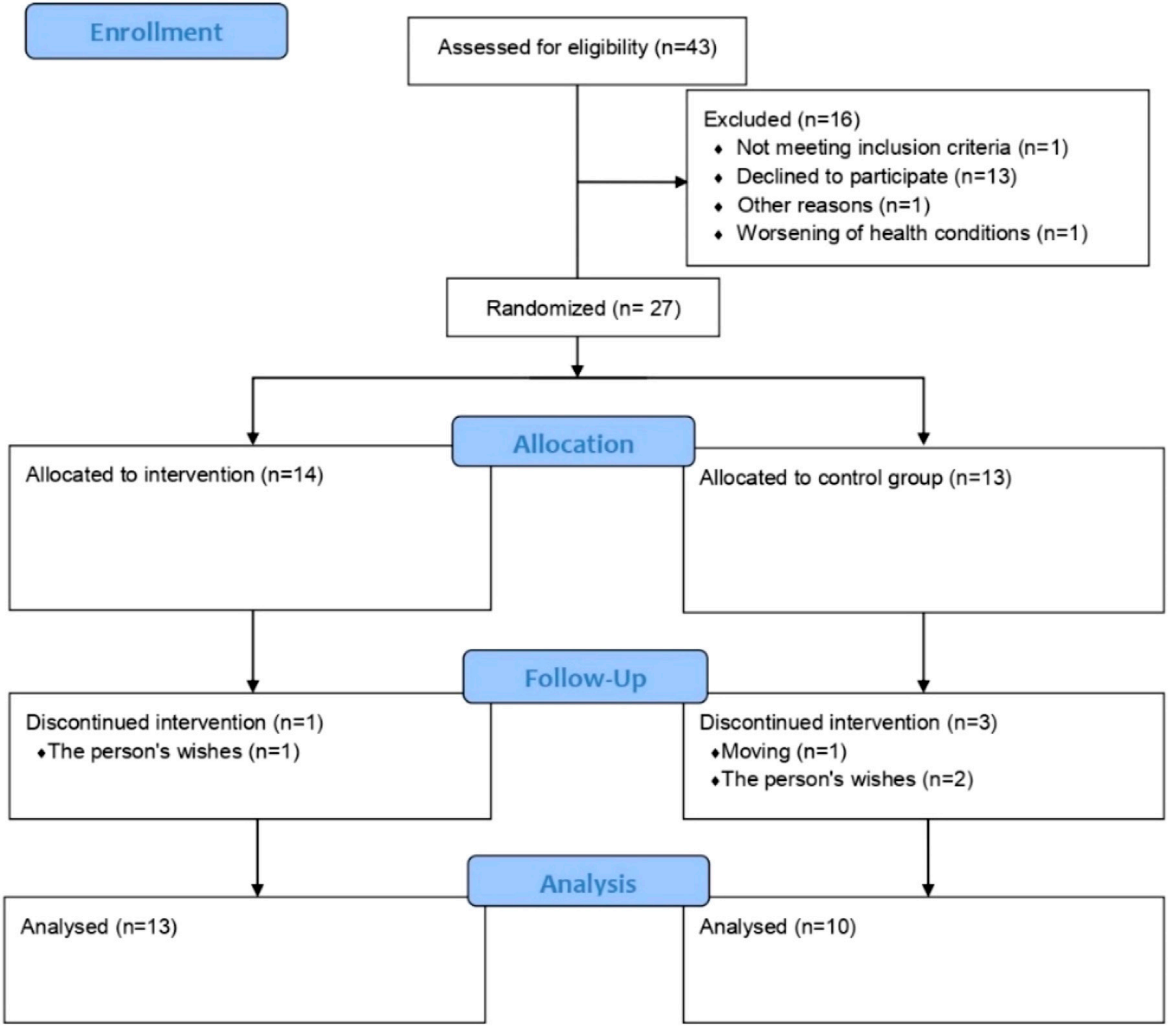
CONSORT flow diagram.

This is an open-label randomized controlled trial. A CONSORT flow diagram of the study is shown in Figure 1. We recruited healthy older adults by placing a study recruitment advertisement in a local information magazine distributed mainly in Sendai City, and received 43 applications. The eligibility criteria were (1) age 65–74 (at the time of registration), (2) Those whose Activities of Daily Living (ADL) have not impaired (able to live independently), and (3) music beginners (those who had never learned music or played a musical instrument other than in school music classes, and those who had never played music or a musical instrument by themselves). Exclusion criteria were (1) a history of dementia, neurological disease, or mental illness and (2) severe visual or hearing impairment. The baseline examination included a cognitive function test (MMSE, Wechsler Memory Scale Logical Memory, Digit Span, Trail Making Test, Stroop Task, Verbal Fluency Task) and psychological health questionnaire (The Profile of Mood States 2nd Edition, Geriatric Depression Scale, Generalized Self-Efficacy Scale, Interpersonal Reactivity Index, Satisfaction With Life Scale) administered to 27 participants. Cognitive and psychological assessments were conducted using paper-and-pencil tests. Participants were then randomly divided into intervention and control groups based on computer-generated random numbers. The intervention group received in weekly 90-minute sessions with the instrument for 16 weeks. The control group was instructed to go about their normal lives without starting the instrument during the intervention period. During the 16-week intervention, one dropout occurred in the intervention group and three dropouts occurred in the control group. After the intervention, all participants underwent the same first baseline examination again. Pre- and post-intervention examiners were not blinded to group assignment. In addition, participants were aware of which group they were assigned to, but control group participants did not know the details of the intervention group’s program.

### 2.2 Intervention

The music sessions for the intervention group were conducted in a university laboratory where the instruments were set up. Sessions consisted of 4–5 participants supervised and lead by one instructor, and the instruments used by the participants were electronic drums, electronic bass guitar, and electronic keyboards. Instrument selection was based on participation preference. The instructor was in charge of melody on the electronic piano who had more than 10 years of experience playing an musical instrument, could play the piano, and had no musical credentials. He used a metronome to adjust the melody to the appropriate tempo and asked the participants to play along with that tempo and melody. The instructor also answered questions from the participants about how to play and read music, and provided feedback on what was good about the participants’ performances. The participants were assisted by students familiar with each of the instruments. The content of the music group session intervention is shown in Table 1. In the first month, instructors taught participants how to read easy-level sheet music and play instruments in person, using guidance materials. During the lecture on how to play the instruments, the instructor demonstrated how to play each instrument, and the participants played the instruments with the help of the instructor. Participants started out by playing one note to a nursery rhyme melody played by the instructor. In the second month, the number of notes was increased and the tempo was raised, and a nursery rhyme song session was conducted. Participants learned from the instructor the following instruments: keyboards (chords, sharps, and flats), drums (how to use snare drum, hi-hat, and bass drum), and guitar (how to move the fingers of the left hand). Participants also had group sessions of short passages of Japanese pop music. From the third month, they practiced a song to be performed at the final presentation. At the end of the fourth month, they were able to play an entire song from the pop and rock genres (“Let it Be,” “Sukiyaki,” etc.,) for the final presentation. Throughout the intervention period, participants in the intervention group were instructed to not practice at home. The control group did not participate in music sessions and was instructed not to start any new instruments or musical activities during the 4 months.

**TABLE 1:** Music group session intervention content.

Month	Activity
1st month	⋅ Determination of musical instruments:
	- Keyboard/Drums/Bass guitar
	⋅ Basic Lecture:
	- How to hold the instruments
	- How to read sheet music
	⋅ Starting with playing a single note:
	- Individual practice/Group session (nursery rhymes: “Frog Song,” Twinkle Twinkle Little Star,” etc.,)
2nd month	⋅ Learning Instruments:
	- Keyboard: Chords, Sharps (#) and Flats (♭)
	- Drums: Multiple drum types (bass drum/snare drum/Hi-hat)
	- Bass guitar: How to move the fingers of the left hand
	⋅ Group music session:
	- Short passages of Japanese pop music/nursery rhymes
3rd and 4th month	⋅ Group music session:
	- Short passages of Japanese pop music/nursery rhymes
	- Practice of the final presentation from the beginning to the end of the song.
	⋅ Final presentation
	- “Let it Be”, “Sukiyaki”etc.,

### 2.3 Measurement

We conducted cognitive and psychological function test. We used the MMSE as a screening cognitive function test (Folstein et al., 1975; Sugishita et al., 2016). The maximum score is 30, with a cutoff of 23/24. In this study, the MMSE was used before and after the intervention for the purpose of measuring overall cognitive function.

We used the Wechsler Memory Scale Logical Memory (Wechsler, 1987) to examine verbal memory. Participants had to recall each story immediately (WMS-LM I, immediate recall) and 30 min later (WMS-LM II, delayed recall).

Digit Span was used to assess short-term and working memory (Wechsler, 1997). In the Digit Span Forward (DS-F) and Digit Span Backward (DS-B), participants answer the numbers said by the examiner in the same or opposite order.

Trail Making Test (Reitan, 1992) was used to evaluate executive function, among other things, and consists of two parts. In Part A, participants connect 25 numbers on a line in sequence as quickly as possible. In part B, participants connect numbers and letters alternately, in sequence, as fast as possible. The time it takes to connect all the numbers is measured, and the shorter the time to completion, the better the performance. ΔTMT evaluates cognitive flexibility.

Stroop Task (Hakoda and Sasaki, 1990) was used to measure executive function, inhibitory control, and selective attention. This task consists of four tasks in total. We administered two control tasks (a word–color task and a color–word task), a reverse Stroop task, and a Stroop task. In each task, the participants were instructed to complete as many tasks as possible in 60 s. In the current study, a Stroop for groups was used and the variable was the number of correct responses rather than reaction time. The following was used as a reference for the calculation of Stroop interference (Hakoda and Sasaki, 1990).

$$\begin{array}{ll} \text{Reverse Stroop Interference}= & \left(\text{number of correct responses in the word}-\text{color task}-\text{number of correct responses in the reverse Stroop task}\right)\;/\;\\ & \left(\text{number of correct responses in the word}-\text{color task}\right)\times 100 \end{array}$$

$$\begin{array}{ll} \text{Stroop Interference}= & \left(\text{number of correct answers in the color}-\text{word task}-\text{number of correct answers in the Stroop task}\right)\;/\;\\ & \left(\text{number of correct answers in the color}-\text{word task}\right)\times 100 \end{array}$$

Verbal Fluency Task (Ito et al., 2004) was used to evaluate verbal function. There were two types of tasks—letter fluency, in which participants said words beginning with a specific letter (e.g., a), and category fluency, in which they said words from a specified category (e.g., fruits, animals). In each test, the more words that were expressed, the better the performance.

Psychological function tests were conducted with five questionnaires. The Profile of Mood States 2nd Edition (POMS 2) (Konuma et al., 2015) was used to investigate mood states. This instrument consists of seven subscales—Anger–Hostility (AH), Confusion–Bewilderment (CB), Depression–Dejection, Fatigue–Inertia (FI), Tension–Anxiety (TA), Vigor–Activity (VA), and Friendliness (F)—and the Total Mood Disturbance (TMD), which represents the overall negative mood state, to assess mood state over a given time frame. Higher TMD scores indicate a more negative mood. TMD scores are calculated as follows.

$$\text{TMD}=\left(\text{AH}+\text{CB}+\text{DD}+\text{FI}+\text{TA }–\text{ VA}\right)$$

The Geriatric Depression Scale (Sugishita et al., 2017) was used to evaluate severity of depressive symptom. This is a typical scale for measuring depression in older adults and consists of 15 items. Higher scores indicate stronger symptoms of depression.

We conducted the Generalized Self-Efficacy Scale (Sherer and Adams, 1983) to measure an individual’s general self-efficacy for events that occur in a variety of everyday situations. Higher scores indicate higher self-efficacy.

Interpersonal Reactivity Index (Davis, 1980) was used to evaluate to empathy. This is the most widely used multidimensional empathy scale, employing four subscales: empathic concern, perspective-taking, personal distress, and fantasy.

Satisfaction With Life Scale (Diener et al., 1985) was used to measure life satisfaction and subjective wellbeing using a 7-point scale. A higher score indicates a higher level of happiness.

### 2.4 Statistical analysis

In the intervention and control groups, demographic data and the respective pre-intervention cognitive function test and questionnaire scores were compared using the unpaired *t*-test and x^2^ test to assess group homogeneity at pre-intervention. As we have confirmed data normality by Shapiro-Wilk test, a two-way repeated measures ANOVA was conducted using group (intervention and control) as a between-participants factor and time (pre- and post-intervention) as a within-participants factor to determine the effects of the music session intervention on behavioral measures. If the interaction was significant, *post hoc* tests with Bonferroni correction were performed. All analyses were considered statistically significant if *p* < 0.05. Data were analyzed using SPSS Windows version 25 (IBM Corp., Armonk, NY, United States).

### 2.5 Ethical considerations

This study was approved by the Ethics Committee of the Graduate School of Medicine at Tohoku University (2023-1-658) and is registered with UMIN-CTR (UMIN000051152). All participants provided verbal and written informed consent.

## 3 Results

### 3.1 Demographics

There were no significant differences in baseline testing for both groups in terms of age, educational history (Table 2). There were no significant differences in the results of psychological indices and cognitive function tests at baseline between the two groups. It noted that all participants scored 24 or higher in the MMSE, indicating they had no cognitive impairment at both baseline and endpoint (Sugishita et al., 2016).

**TABLE 2:** Demographics.

	Experimental group		Control group		t/χ^2^
	Mean	SD	Mean	SD	
Age	69.00	3.06	69.60	2.41	0.615
Education	14.31	1.60	13.80	2.20	0.528
Men/Women	7/6		5/5		0.855

At the time of the first group assignment, we investigated whether there were any differences in the profiles of the two groups.

### 3.2 Cognitive function results

**FIGURE 2:**
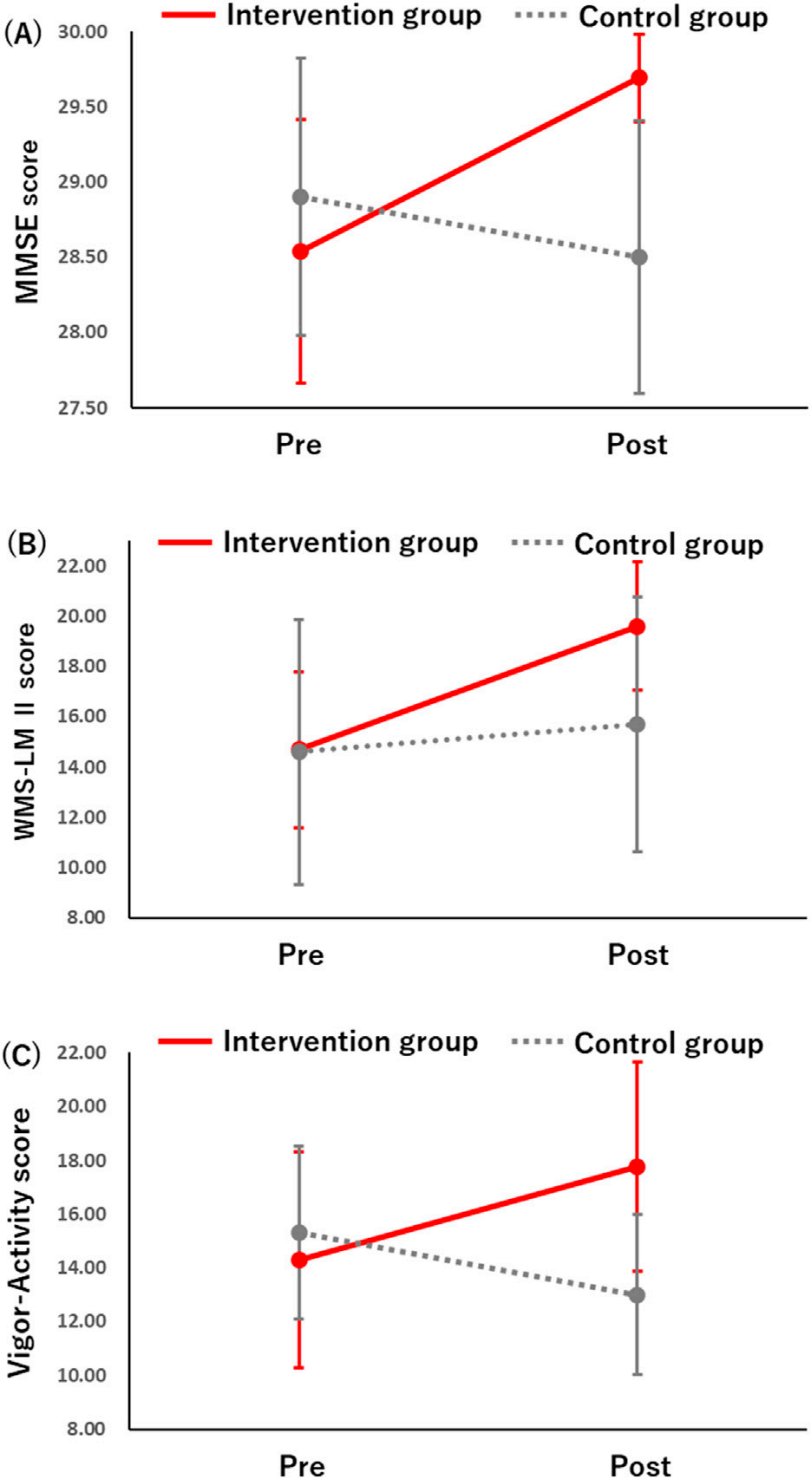
Effect of the music session group-by-time point interaction on **(A)** MMSE, **(B)** WMS-LM II, **(C)** POMS 2 (VA) scores.

The values of cognitive function are shown in Table 3. Among them, significant interactions were revealed in MMSE and WMS-LM II (Figure 2). Regarding MMSE total scores, there was no significant main effect of time (F_(1,21)_ = 1.052, *p* = 0.317, $\eta_{p}^{2}$ = 0.048) and group (F_(1,21)_ = 1.951, *p* = 0.218, $\eta_{p}^{2}$ = 0.071), while there was a significant group × time interaction (F_(1,21)_ = 4.471, *p* = 0.047, $\eta_{p}^{2}$ = 0.176). At baseline, there was no difference in MMSE total scores between the two groups (*p* = 0.541). In addition, MMSE scores increased significantly in the intervention group before and after the intervention (*p* = 0.027), while there was no significant change in MMSE scores in the control group (*p* = 0.477). In the WMS-LM II, there was a significant main effect of time (F_(1,21)_ = 12.170, *p* = 0.002, $\eta_{p}^{2}$ = 0.367) but no main effect of group (F_(1,21)_ = 0.735, *p* = 0.401, $\eta_{p}^{2}$ = 0.034). There was also a significant group × time interaction in the WMS-LM II (F_(1,21)_ = 4.903, *p* = 0.038, $\eta_{p}^{2}$ = 0.189). The intervention group showed a significant increase in WMS-LM II scores before and after the intervention (*p* < 0.001), while the control group showed no significant change in WMS-LM II scores (*p* = 0.406). There was no difference in WMS-LM II scores between groups at baseline (*p* = 0.972), and no significant difference between groups post-intervention (*p* = 0.114). These results indicate that verbal memory improved with intervention. No significant interactions or differences were found for other measures of cognitive function.

**TABLE 3:** Cognitive function results.

	Experimental group				Control group				Group × time
	Pre-test		Post-test		Pre-test		Post-test		
	Mean	SD	Mean	SD	Mean	SD	Mean	SD	p-value
MMSE (points)	28.54	1.45	29.69	0.48	28.90	1.29	28.50	1.27	0.047
WMS-R (points)									
WMS-LM Ⅰ	20.23	4.44	23.85	4.54	19.80	8.01	20.10	5.17	0.124
WMS-LM Ⅱ	14.69	5.12	19.62	4.21	14.60	7.38	15.70	7.12	0.038
DS (points)									
DS-F	9.54	2.40	10.08	1.55	10.10	2.18	9.70	2.75	0.247
DS-B	5.77	1.59	6.00	1.68	5.80	1.32	5.90	2.13	0.862
TMT (s)									
TMT-A	45.58	9.17	37.99	7.37	41.16	7.62	38.52	11.30	0.258
TMT-B	82.10	27.45	75.52	30.15	67.89	16.12	75.04	33.24	0.332
ΔTMT	36.61	30.06	37.53	30.81	26.73	20.56	36.52	23.11	0.497
Stroop Task (%)									
Reverse Stroop Interference	14.10	7.97	16.89	9.30	8.89	5.89	11.49	4.67	0.953
Stroop Interference	16.75	19.17	18.07	13.41	12.51	7.47	19.60	9.50	0.517
VFT (points)									
VFT Letter	27.54	8.90	33.62	8.27	29.30	11.28	31.40	9.41	0.193
VFT Category	39.46	9.64	40.85	7.13	44.40	9.41	43.20	6.66	0.324

All scores were presented as points. Data were shown as mean ± SD. WMS-R, wechsler memory scale revised; WMS-LM Ⅰ, Wechsler Memory Scale Logical MemoryⅠ; WMS-LM Ⅱ, Wechsler Memory Scale Logical Memory Ⅱ; DS, digit span forward; DS-F, digit span forward; DS-B, digit span backward; TMT, trail making test; TMT-A, Trail Making Test part A; TMT-B, Trail Making Test part B; ΔTMT, Trail Making Test part B- Trail Making Test part A; VFT, verbal fluency task.

### 3.3 Psychological function results

The values of psychological functions are shown in Table 4. Among them, significant interactions were revealed in POMS2 VA (Figure 2). In the VA, there was no significant main effect of time (F_(1,21)_ = 0.178, *p* = 0.677, $\eta_{p}^{2}$ = 0.008) and group (F_(1,21)_ = 0.929, *p* = 0.346, $\eta_{p}^{2}$ = 0.042), while there was a significant group × time interaction in VA scores (F_(1,21)_ = 4.381, *p* = 0.049, $\eta_{p}^{2}$ = 0.173). There was no difference between groups on the baseline VA (*p* = 0.690) and no significant difference between groups on the post-intervention VA (*p* = 0.054). There was also an increase in scores for the intervention group and a decrease in scores for the control group, but the change in each score was not statistically significant (intervention group, *p* = 0.070; control group, *p* = 0.279). These results indicate a different pattern of change in VA scores between the intervention and control groups before and after the intervention. There were no significant differences in other measures of psychological function.

**TABLE 4:** Psychological function results.

	Experimental group				Control group				Group × time
	Pre-test		Post-test		Pre-test		Post-test		
	Mean	SD	Mean	SD	Mean	SD	Mean	SD	p-value
POMS 2 (points)									
AH	6.92	6.38	6.54	5.97	5.30	5.33	7.20	8.40	0.428
CB	13.85	5.01	13.46	5.78	12.80	4.92	12.40	4.20	0.993
DD	8.62	7.47	7.15	7.39	7.60	7.14	7.80	5.75	0.534
FI	4.77	4.71	4.54	3.20	7.30	3.92	5.60	3.98	0.399
TA	11.77	5.96	11.23	5.80	14.40	6.88	13.00	6.38	0.740
VA	14.31	6.65	17.77	6.41	15.30	4.50	13.00	4.16	0.049
F	12.00	3.49	13.23	3.61	10.00	3.46	10.80	2.90	0.812
TMD	31.62	29.42	25.15	28.85	32.10	19.32	33.00	28.23	0.464
GDS (points)	4.08	3.71	3.15	3.51	3.30	2.98	4.50	4.14	0.084
GSES (points)	52.08	11.71	53.00	13.96	54.10	8.37	55.20	7.74	0.948
IRI (points)									
PD	12.92	3.82	14.00	4.42	14.00	3.27	14.00	2.16	0.479
EC	18.62	3.40	18.77	2.98	18.40	2.41	19.20	2.90	0.479
PT	15.54	3.04	16.00	2.04	14.90	3.98	15.40	3.89	0.977
FS	13.23	5.13	14.85	5.08	12.50	5.84	13.60	4.55	0.627
SWLS (points)	23.62	5.56	22.77	5.05	22.00	3.27	19.80	4.64	0.335

All scores were presented as points. Data were shown as mean ± SD. POMS 2, The Profile of Mood States 2nd Edition; AH, Anger-Hostility; CB, Confusion-Bewilderment; DD, Depression-Dejection; FI, Fatigue-Inertia; TA, Tension-Anxiety; VA, Vigor-Activity; F, friendilness; TMD, total mood disturbance; GDS, geriatric depression scale; GSES, Generalized Self-Efficacy Scale; IRI, interpersonal reactivity index; PD, personal distress; EC, empathic concern; PT, perspective taking; FS, fantasy scale; SWLS, satisfaction with life scale.

## 4 Discussion

We investigated the effects of a 16-week group music intervention on cognitive and psychological functions in healthy older adults who had never played a musical instrument. The results showed that cognitive function improved, with overall cognitive function and verbal memory improving because of the session-based intervention. In psychological functioning, the intervention had an effect on some indicators. The improvement in total MMSE scores between before and after the intervention suggests that the session-based format improved overall cognitive function in healthy older adults. These results are consistent with reports of MMSE score improvements in individuals with declining cognitive impairment, including a drum session intervention for patients with dementia and a group music intervention with rhythmic movement for nursing home residents (Miyazaki et al., 2020; Kim and Kang, 2021). Other studies have suggested the impact of multimodal activities on cognitive improvement (Hars et al., 2014; Nishiguchi et al., 2015). Playing a musical instrument itself is a multimodal activity that includes auditory, visual, and tactile senses (Zatorre et al., 2007; Lappe et al., 2008). Furthermore, during musical sessions, musicians need to use both auditory and visual cue modalities to enhance synchronization with others (Goebl and Palmer, 2009; Kawase, 2014; Bishop and Goebl, 2015). Tasks that need synchronization with others like as a music session require complex cognitive processing that involves a wider range of brain regions (Chen et al., 2006; Pollok et al., 2009; Hove et al., 2013), and therfore are assumed to place a higher multi-modal cognitive load than musical instrument performance, which does not require synchronization. Therefore, music sessions may have influenced improvements in overall cognitive function, as measured by the MMSE, even in healthy older adults without cognitive impairment. In the present study, significant improvements were found in the MMSE and in the WMS-LM II, suggesting a positive effect of music sessions on verbal memory. This is consistent with a previous study of keyboard harmonica training that reported improved WMS-LM II scores (Guo et al., 2021), suggesting that the activity of singing melodies with the keyboard harmonica is a kind of verbal memory practice. Although the participants did not sing during this study’s music sessions, it is possible that recalling the lyrics as they played familiar songs influenced the improvement in verbal memory (Wang, 2007; Lök et al., 2019; Cunningham et al., 2019). The keyboard harmonica training is known to correlate the neural efficiency of lPu-rSTG connectivity with improved verbal memory performance (Guo et al., 2021), and such neural efficiency improvement may have occurred in the background of the improved verbal memory in the current study. Professional musicians are also known to have a smaller range of fMRI BOLD signal activation during performance than amateur musicians, a view that long-term music training is associated with increased efficiency and reduced effort (Lotze et al., 2003). This suggests that musicians become more efficient at playing their instruments through long-term training, and may provide evidence to support the “neural efficiency theory” proposed by Haier (Haier et al., 1988; Haier et al., 1992a; Haier et al., 1992b). In addition, in the present study, a trend of improvement was observed in the POMS 2’s VA factor in psychological functioning. In a previous study of piano lessons, improvement in the mood state (FI factor) among healthy older adults was reported (Seinfeld et al., 2013). Music therapy for hospitalized patients improved scores on the POMS TMD and TA, AH, FI, and CB factors (Cassileth et al., 2003). While a certain mood-improving effect has been reported with individual performances in the literature, there have been no reports of improvement in VA; this is the first time it has shown a tendency to improve with music sessions. Although there have been other reports of music therapy improving mood and depression in hospitalized patients (Martí et al., 2023), there have been few previous studies on the effects of music sessions on positive mood, and the relationship between music sessions and mood should be investigated in the future.

Although some items presented significant improvements in both cognitive and psychological functions, there is a possibility that neuroscience can explain the relationship between psychological and cognitive function improvements. Amyloid accumulation and tau-induced neurofibrillary changes are associated with the development of dementia (Bloom, 2014), and cerebrospinal fluid biomarkers such as amyloid-β42 and phosphorylated tau are known to be useful in the early diagnosis of Alzheimer’s disease. The ratio of tau/Aβ42 could indicate cognitive decline (Fagan et al., 2007; Chen et al., 2019), and regular exercise habits are known to be associated with improved tau/Aβ42 ratios (Hou et al., 2021). However, this ratio is not only related to dementia but also to mood status. In cognitively normal older adults, participants with higher cerebrospinal fluid tau/Aβ42 values have been found to have greater increases in mood disorder scores throughout the one-year follow-up period compared with participants with lower values (Babulal et al., 2016). In patients with mild cognitive impairment, smaller VA scores on the POMS 2 have been associated with more amyloid plaques and tau enrichment in brain regions involved in emotion regulation (Chen et al., 2014). It is also known that in older women at high risk for Alzheimer’s disease, greater negative mood symptoms are associated with higher tau enrichment and greater vitality with lower tau enrichment (Ahmadi et al., 2024). It is also possible that other brain substances were affected in this study’s music sessions, leading to the improvement in cognitive and psychological functions. Dopamine released in the brain is involved in reward system mechanisms and feelings of well-being (Alexander et al., 2021), and is also related to levels of brain-derived neurotrophic factor (BDNF), which is associated with the development of Alzheimer’s disease (Leo et al., 2018). In animal studies, listening to classical music in mice enhanced BDNF signaling, synaptic proteins, and neurogenesis (Cheng et al., 2024). In humans, functional magnetic resonance imaging and positron emission tomography studies have shown that listening to pleasant music increases dopamine activity in the striatum (Zatorre, 2015; Mueller et al., 2015). Other significant differences have also been found in the levels of BDNF in plasma between people who usually play music and those who do not (Minutillo et al., 2021). Although we did not measure tau enrichment or physiological indices of dopamine and BDNF, it is suggested that changes in intermediate indices such as the release and localization of neurotransmitters and proteins in the brain may have occurred behind the improvement in cognitive and psychological functions as phenotypes in this study.

There were no significant improvements in executive functions, as demonstrated by scores on the TMT, Stroop, and verbal fluency tests. Previous studies have reported improved and unimproved executive function depending on the total number of hours, frequency, type, intensity, and difficulty of musical instrument intervention (Seinfeld et al., 2013; Bugos, 2019; MacRitchie et al., 2020; Guo et al., 2021; Wang et al., 2023). Although several studies have reported positive effects with music sessions (Miyazaki et al., 2020; Kim and Kang, 2021), the optimal total number of hours, frequency, and intensity of music session for cognitive benefits remains unclear. Based on previous findings, we implemented weekly 90-minute sessions for 16 weeks (total number of hours is 24 h) in this study; however, our results suggest that enhancing executive function through neuroplasticity may require a greater total number of hours, more frequent sessions or the incorporation of more complex musical activities. In a previous study, an intervention of piano lessons with a high degree of difficulty using both hands significantly improved executive and other functions (Seinfeld et al., 2013). On the contrary, in another previous study, music training in relatively easy keyboard harmonica playing using one hand did not improve executive function (Guo et al., 2021). Other studies have reported that performance music training significantly improves executive function (Bugos, 2019; MacRitchie et al., 2020), but not significantly (Wang et al., 2023). Looking at the effect of playing a musical instrument on executive function in terms of the total number of hours of intervention, previous studies, including the current study, suggest that at least about 40 h or more of musical instrument intervention, including practice time, may be necessary to enhance executive function. In addition, there were no significant improvements in psychological measures other than the POMS 2, but this may be because the baseline scores were originally high given that the sample consisted of healthy older adults.

This study has several limitations, the first of which is using self-reports. The current study is an open-label intervention design and use self-reports to evaluate some effects of intervention. Therefore, there could be bias had occurred and effects results. The second is the relatively small sample size. Although previous studies have investigated the effects of playing musical instruments with sample sizes similar to the current study (Bugos et al., 2007; Seinfeld et al., 2013; MacRitchie et al., 2020), a larger sample size is necessary to increase the robustness of the results. Third, the cognitive and psychological improvements may be attributed to social interactions during music sessions, Therefore, we cannot conclude music sessions cause these improvements in this study. In the future, it will be necessary to add social interaction tasks to the control group and to control for the frequency and content of social interactions by conducting questionnaire surveys. Fourth, the effects of different instruments (keyboard, guitar, and drums) on the cognitive psychological indices in the intervention group may differ, but we were unable to analyze this aspect owing to the small sample size. However, as all instruments use both hands, we believe that bimanual coordination is consistent among the instruments. Finally, we did not directly compare and verify the direct effects of music sessions conducted by multiple people versus playing a single instrument alone, and future research should better clarify the direct effects of music sessions.

In this study, the activities of the music sessions improved cognitive and psychological functions. Although the music (band) session was conducted for 16 weeks with multiple types of instruments, future studies are needed to examine the content and intensity of the music session intervention to investigate what type of music sessions are more effective. In addition, it is expected that future studies of music sessions will collect data on neurophysiological data that will provide evidence to explain the effects of music sessions, thereby clarifying the mechanism of the relationship between music sessions and cognitive and psychological functions. These findings might provide insight into dementia prevention.

## Data Availability

The original contributions presented in the study are included in the article/Supplementary Material, further inquiries can be directed to the corresponding author.
